# Mechanical Properties and Failure Mechanism of Recycled Concrete with Different Coal Gangue Powder Contents

**DOI:** 10.3390/ma18112572

**Published:** 2025-05-30

**Authors:** Xianda Ren, Rui Wu, Yongli Xie

**Affiliations:** School of Highway, Chang’an University, Xi’an 710064, China

**Keywords:** recycled aggregate, coal gangue powder, uniaxial compression, crack percent, failure mechanism

## Abstract

In this study, the mechanical properties and failure characteristics of concrete with 10–20 mm recycled coarse aggregate at 0%, 25%, 50%, 75%, and 100% substitution rates were studied. In addition, the influence of coal gangue powder (CGP) on the mechanical properties of concrete was studied under the dosages of 5%, 10%, 15%, and 20%. The research results show that the peak strength of recycled concrete decreases with the increase in the replacement rate of 10–20 mm recycled coarse aggregate. When the replacement rate is 25%, the decrease in strength is the smallest. When the content of CGP increases from 0 to 20%, the peak strength of recycled concrete increases first and then decreases. When the content of CGP is 15%, the peak strength reaches the maximum value. The peak strength increases slightly. The density of pores and cracks in recycled concrete increases with the increase of 10–20 mm recycled coarse aggregate replacement rate. When the substitution rate exceeds 25%, the proportion of cracks increases by nearly 1.7 times. After adding CGP to recycled concrete, the pore density and crack ratio inside a specimen are significantly reduced. When the CGP content exceeds 15%, the crack ratio tends to be stable. When the CGP content is 15%, the crack ratio is 0.519%, which is 23.5% lower than that of the RAC-25 specimen. When the content exceeds 15%, the crack ratio tends to be stable.

## 1. Introduction

The preparation and production of concrete requires a large amount of mineral resources and freshwater resources, and it is also an important factor in the deterioration of the global environment and climate. In recent years, due to the rapid advancement of urban modernization, a large amount of non-degradable construction waste has been collected in the short term, which puts great pressure on the ecological environment. An estimated 900 million of construction waste are generated every year in Europe, the USA, and Japan, as reported by the World Business Council for Sustainable Development [1]. Therefore, the renewable utilization of construction waste has become a serious challenge facing the world, and the scientific research on recycled concrete has become a hot issue for scholars in various countries [2]. In addition, with the deepening understanding of the resource utilization of coal gangue, the research on the preparation of cementitious materials from coal gangue has become a hot topic.

There are two kinds of interfacial transition zones (ITZs) between the surface of recycled coarse aggregate and hardened mortar in recycled concrete. Among them, the old ITZ exists between the recycled coarse aggregate and the old attached mortar, and the new ITZ exists between the recycled coarse aggregate and the new hardened mortar [3]. The pore distribution characteristics of the new ITZ are related to the initial water content of the RCA and the strength of the RCA source concrete [4]. Guo et al. [5] found that when the replacement rate of recycled coarse aggregate (RCA) and recycled fine aggregate (RFA) was 100%, the compressive strength of recycled concrete was 36%~42% lower than that of natural aggregate concrete (NAC). Omry et al. [6] established the relationship between the physical and mechanical properties of aggregates through comparative tests of recycled aggregates and natural aggregates. Xiao and other scholars [7] showed that when the replacement rate of recycled aggregate was 50%, the compressive strength of recycled aggregate concrete was the highest, but the compressive strength of recycled aggregate concrete was still lower than that of natural aggregate concrete. In addition, scholars [8] also studied the uniaxial tensile test of five groups of recycled concrete prism specimens. The results showed that in the process of tension, recycled aggregate concrete had a great influence on the compressive strength of concrete. Kwon et al. [9] studied the mechanical properties of recycled concrete with different replacement rates. Compared with natural coarse aggregate, recycled coarse aggregate had a high crushing value index, low apparent density, and low bulk density, and its water absorption was higher, about 23 times that of natural coarse aggregate [10].

A large number of studies have shown that the physical and mechanical properties of recycled aggregate have significant effects on the failure characteristics and cracking mode of concrete. Reference [11] used digital image correlation technology to explore the failure process of recycled block concrete under compression loading. The results showed that when the strength of new and old concrete was close, the joint surface of new and old concrete was not an obvious weak part in the uniaxial compression test. References [12,13] found that when the strength of waste concrete was lower than that of new concrete, the increase in the replacement rate of waste concrete block would lead to a decrease in the splitting tensile strength of recycled block concrete. When the waste concrete block was broken off the recycled concrete block, the inevitable micro-cracks and the interface between the new and old concrete could become seepage channels, which had adverse effects on the mechanical characteristics and durability of the recycled concrete [14]. In Reference [15], crack propagation was modeled by using fracture mechanics, simulation, and machine learning (ML), and experiments were carried out on various famous crack image datasets. Wang et al. [16] found that the shear strength of natural concrete (MNC) was the highest, followed by simulated recycled concrete (MRC). The shear cracks of MNC first appeared in the interface transition zone (ITZ) and expanded, resulting in shear failure. The above research results show that the existence of old mortar makes the interface transition zone more porous, which is the fundamental reason for the deterioration of recycled concrete performance and durability [13,17].

Coal gangue powder is a fine material created by crushing and grinding the solid waste generated during coal mining and washing. It can be used as a mineral admixture in concrete and recycled concrete, which reduces the amount of cement required, improves workability, and enhances both compressive strength and durability. Wu et al. [18] verified the reliability of using recycled CG powder to develop sustainable ultra-high-performance concrete (UHPC), and its performance was assessed. The addition of CG powder was found to reduce the early compressive strength of UHPC while enhancing its strength at 28 days. All UHPC samples containing CG powder met the minimum cube strength requirement of 150 MPa for UHPC at 28 days. Li et al. [19], through single-factor experiments, orthogonal experiments, and microscopic performance tests, along with theoretical analysis, determined the optimal mix ratio of CGP-RAC, as well as the factors affecting its mechanical properties, the behavior of strength development, and the underlying influence mechanisms. The results indicated that both the compressive strength and splitting tensile strength of CGP-RAC decreased as the substitution rate of CGP-RAC increased. Zhu et al. [20] found that as the CGP content of crushed glass powder (CGP) increased, the concrete strength initially decreased but improved in the later stages.

Under different gradations, the particle size of the aggregate is different, and its composition is also different [21]. When the particle size is 5–10 mm, the aggregate is mainly composed of broken stones and old mortar blocks. When the particle size is 10–20 mm, the aggregate is composed of broken stones, gravel–old mortar blocks, and old mortar blocks. To facilitate the observation and analysis of the failure characteristics and failure mechanism of recycled concrete, this paper selects recycled concrete aggregate with 10–20 mm aggregate and analyzes the mechanical properties of recycled concrete under different aggregate replacement rates. At the same time, digital image processing technology is used to analyze the distribution characteristics and proportional relationship of failure cracks. On this basis, the compressive strength and failure characteristics of recycled concrete with different CGP contents are analyzed.

## 2. Experiment

### 2.1. Material and Mix Ratio

The material used in this experiment was recycled concrete after crushing at a construction site. The aggregate was washed to remove impurities, such as residual bricks and wood blocks. The aggregate was dried and screened according to the particle size distribution. It was divided into three groups according to 0–5 mm, 5–10 mm, and 10–25 mm. Several trial concrete specimens produced with the RCA were tested, and the results showed that both data discreteness and material strength can meet the test requirements.

The cement used in this experiment was P.II42.5R-grade cement with relatively stable properties. The specific surface area of the cement was 350 m^2^/kg, and the density was 3.12 g/cm^3^. The CGP was the first type of calcareous rock coal gangue, crushed with a density of 2.90 g/cm^3^ and a specific surface area of 468 m^2^/kg. The composition of the materials is shown in Table 1.

**Figure 1:**
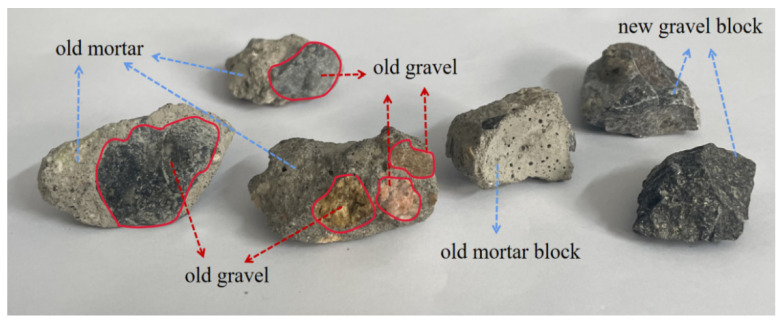
Composition of 10–20 mm recycled aggregate.

In recycled aggregate, the proportion of old mortar attached to the surface of different graded aggregates is also different, and the proportion of old mortar has a significant impact on the mechanical properties of recycled concrete. Figure 1 shows the recycled aggregate selected in the experiment. Through observation, the recycled aggregate can be divided into three parts, including gravel, old mortar block, and mortar–gravel block, in which the mortar part has higher porosity. In the four-stage grading of coarse aggregate, the content of old mortar in medium stone, large stone, and boulder is higher, which can seriously reduce the performance of recycled concrete. Therefore, the present study focused on the influence of the particle size distribution of the secondary aggregate grading concrete on the mechanical properties of recycled concrete. The basic properties of the coarse aggregates are shown in Table 2.

For the coarse aggregate with a particle size ranging from 10 mm to 20 mm, five kinds of recycled aggregate with different replacement rates (r = 0%, 25%, 50%, 75%, and 100%) were considered. And the corresponding specimen groups were named NAC, RAC-25, RAC-50, RAC-75, and RAC-100, respectively. At the same time, this study also analyzed the influence of CGP on the mechanical properties of concrete under the dosage of 5%, 10%, 15%, and 20%. Therefore, the experimental groups were set up as NAC-C-5, NAC-C-10, NAC-C-15, and NAC-C-20. In addition, for recycled concrete specimen RAC-25 with a substitution rate of 25%, the mechanical properties of RAC-25 with 5%, 10%, 15%, and 20% CGP were studied. The experimental groups were RAC-25, RAC-25-C-5, RAC-25-C-10, RAC-25-C-15, and RAC-25-C-20, where C represents the incorporation of CGP. The specific mix ratio of the test piece is shown in Table 3. In addition, extra water was added, and the volume of this added water was equal to the mass of the recycled aggregate in the mix ratio multiplied by the net water absorption (the difference between the measured water absorption and the water content listed in Table 2; when r = 100%, the extra water is 19.7 kg).

### 2.2. Test Scheme

**Figure 2:**
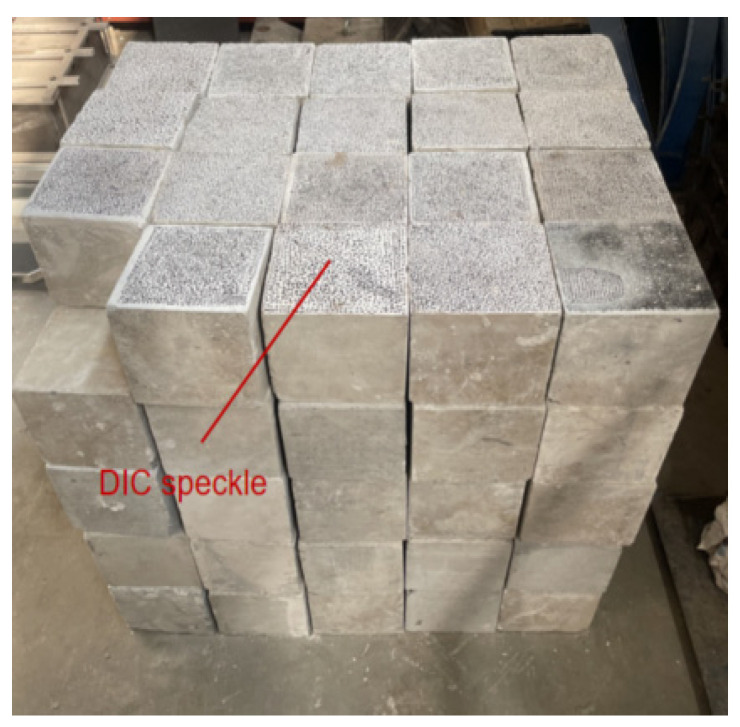
Concrete specimens.

As described in the relevant specifications, the cube samples with dimensions 150 mm × 150 mm × 150 mm, in height × length × width, were prepared for the uniaxial compression test and splitting test, as shown in Figure 2.

The designed concrete strength was 30 MPa. In order to prevent the shrinkage crack of the concrete specimen from affecting the test analysis, the specimen was subjected to standard curing and wet curing.

**Figure 3:**
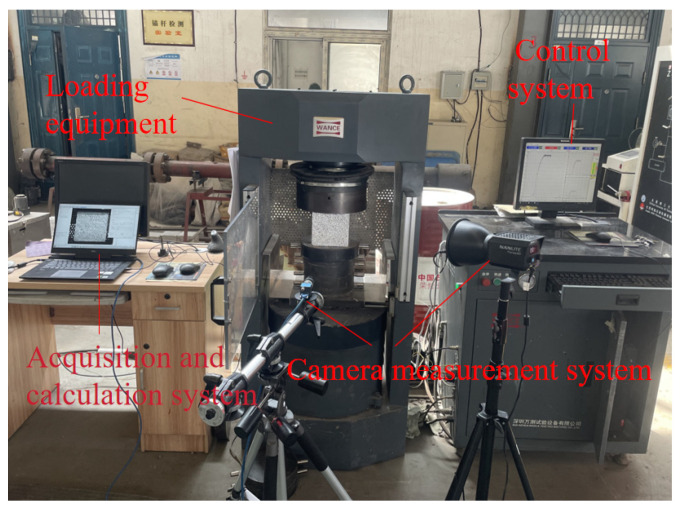
Test equipment.

Figure 3 illustrates that this test relied on the electro-hydraulic servo rock triaxial apparatus. The recycled concrete specimens were placed in the center of the concrete pressure testing machine pedestal, with the top surface of the load plate aligned vertically with the top of the concrete specimen. For the split tensile tests, two steel rods were placed at the upper and lower portions of the concrete to ensure that the steel rods were positioned at the center of the concrete. The loading was performed using a displacement-controlled method with a loading rate of 0.05 mm/min.

## 3. Results

### 3.1. Uniaxial Compressive Strength

**Figure 4:**
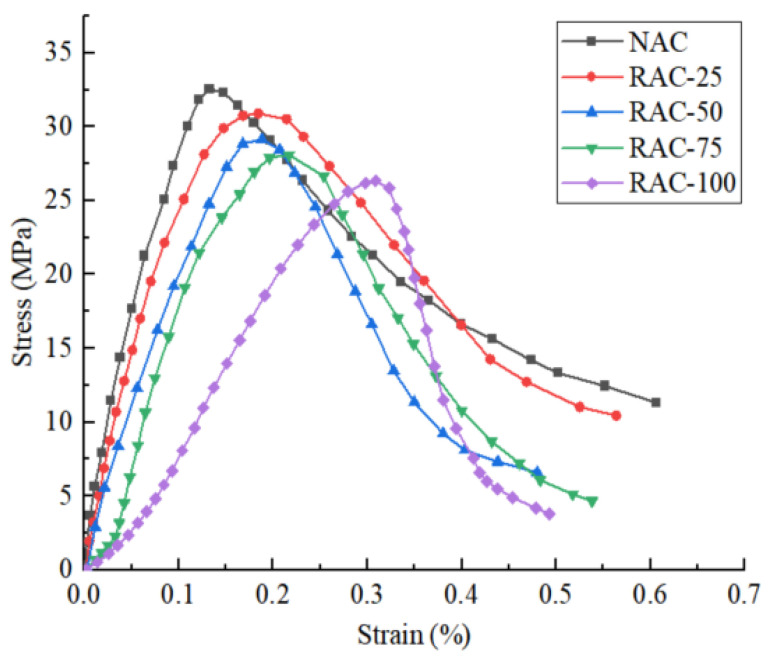
The stress–strain curves of concrete specimens with different recycled aggregate substitution rates.

**Figure 5:**
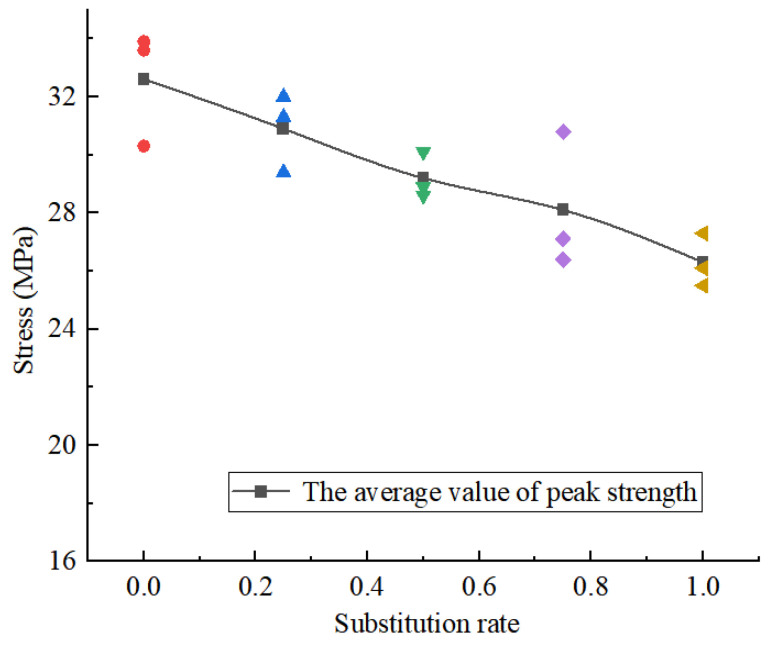
The peak strength of concrete specimens with different recycled aggregate substitution rates.

Figure 4 shows the stress–strain curves of the concrete specimens with different replacement rates of 10–20 mm coarse aggregate. Figure 5 presents the peak strength of concrete specimens at various rates of recycled aggregate substitution.

It can be seen that the process from load bearing to failure of the specimen can be divided into four stages [22], namely, the compaction stage, the elastic deformation stage, and the plastic deformation stage, among which the plastic deformation stage is further divided into the crack damage stage and the failure stage. The peak strength changes with the increase in the regenerated coarse aggregate replacement rate. The peak strength of the sample decreases gradually with the increase in the replacement rate of recycled coarse aggregate. When the replacement rate increases from zero to 100%, the compressive strength of the concrete sample decreases by about 19.3%. With the increase in the substitution rate, the elastic modulus decreases. When the substitution rate reaches 100%, the elastic modulus is the lowest. The plasticity of the concrete specimens decreases with the increase in the substitution rate. When the regenerated aggregate replacement rate is 75% and 100%, the sample has a short compaction process in the uniaxial compression process, and the compaction strain is about 0.05%. When the substitution rate is lower than 75%, no obvious compaction process is found during compression.

**Figure 6:**
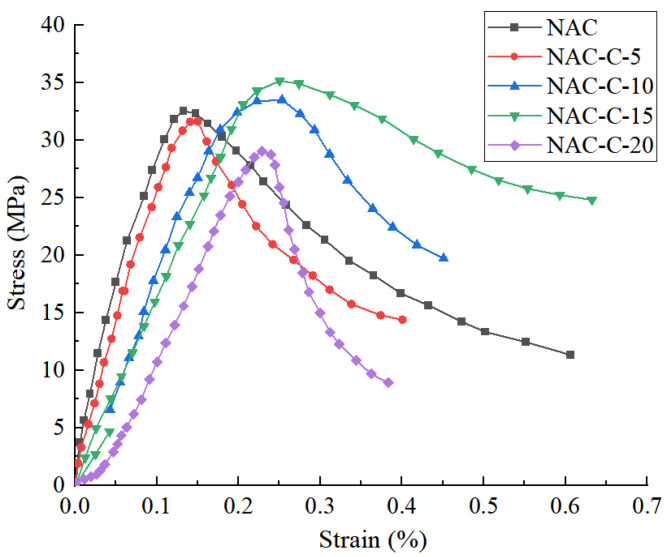
The stress–strain curves of NAC specimens with different CGP contents.

Figure 6 shows the stress–strain curves of the original concrete NAC under different CGP contents.

**Figure 7:**
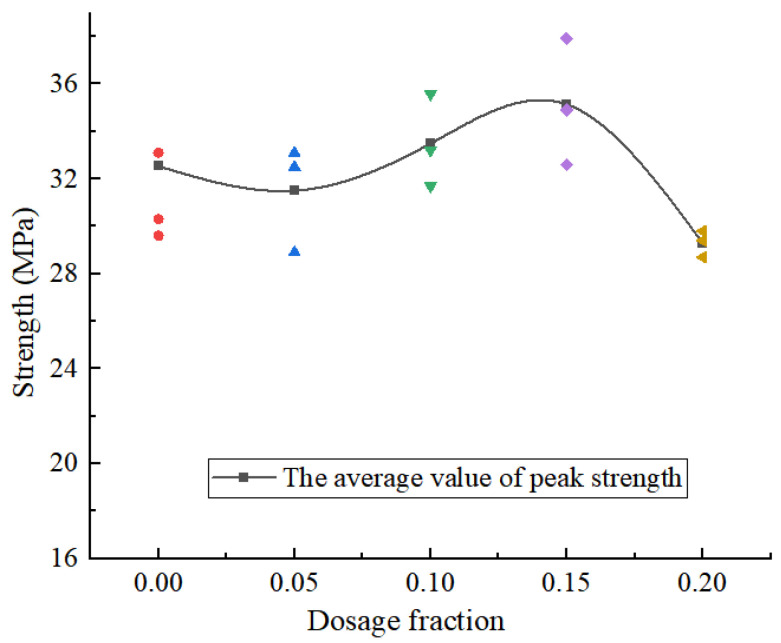
The peak strength of NAC samples under different CGP contents.

It can be seen that when the content of CGP is less than 15%, the specimen only exhibits the stages of elastic deformation, crack propagation, damage failure, and sustained loading, while the compaction stage is not clearly evident. When the content of CGP increases from 0 to 5%, the peak strength of the concrete decreases slightly, with a decrease of only 3.2%. With the increase in the CGP content, when the content reaches 15%, the peak strength of the concrete is the largest, and the specimen with CGP is increased by 8.1%. After that, with the increase in the CGP content, the peak strength of the concrete decreases sharply and is 16.6% lower than the maximum value. In Figure 7, it can be seen that the optimal dosage of CGP is 15%, and the peak strength of the concrete reaches the maximum value. In addition, when the dosage reaches 20%, the value is lower than that of the concrete without CGP.

**Figure 8:**
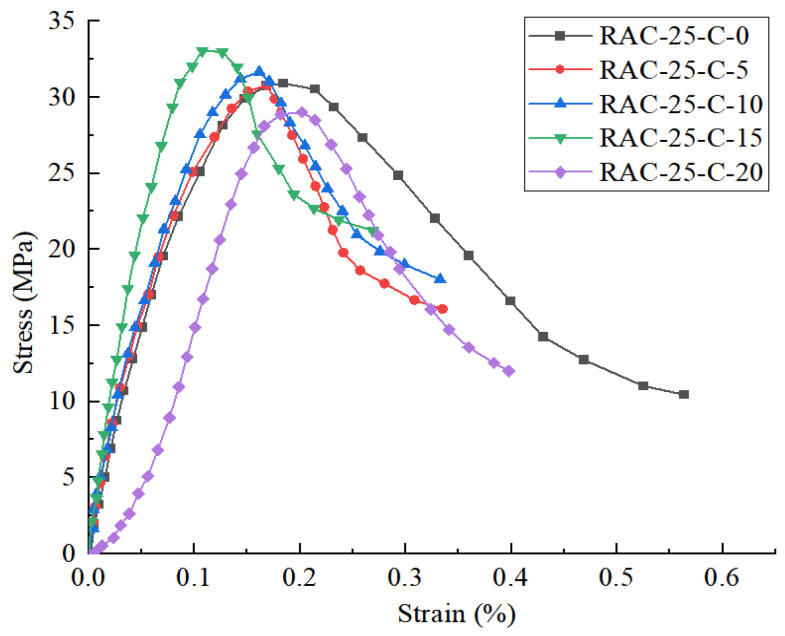
The stress–strain curves of RAC-25 specimens with different CGP contents.

**Figure 9:**
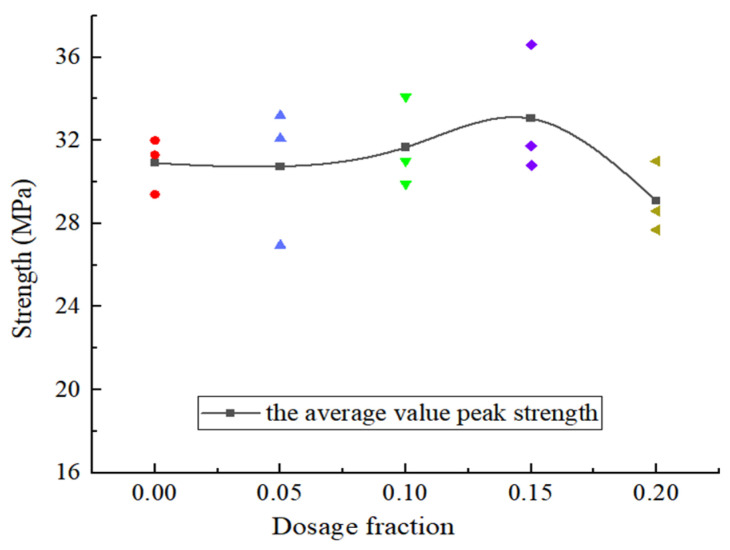
The peak strength of RAC-25 specimens with different CGP contents.

When the replacement rate of the recycled aggregate reaches the optimal value of 25%, the stress–strain curve and peak strength of the recycled concrete with varying CGP contents are illustrated in Figure 8 and Figure 9, respectively.

It can be seen that when the content of CGP is 0, 5%, and 10%, before reaching the peak strength, the curve characteristics of the three specimens in the elastic stage and the crack propagation stage are basically the same. At this time, the first half of the three curves almost coincide, while in the second half of the plastic deformation, the plasticity of specimen RAC is the largest. When the content is less than 15%, the recycled concrete shows the compaction stage, and the peak strength of the specimen increases slowly with the increase in the content. When the content is 15%, the peak strength reaches the maximum value, and the peak strength is increased by 6.9%. It can be seen that CGP has little effect on the peak strength of RAC-25.

### 3.2. Splitting Tensile Strength

**Figure 10:**
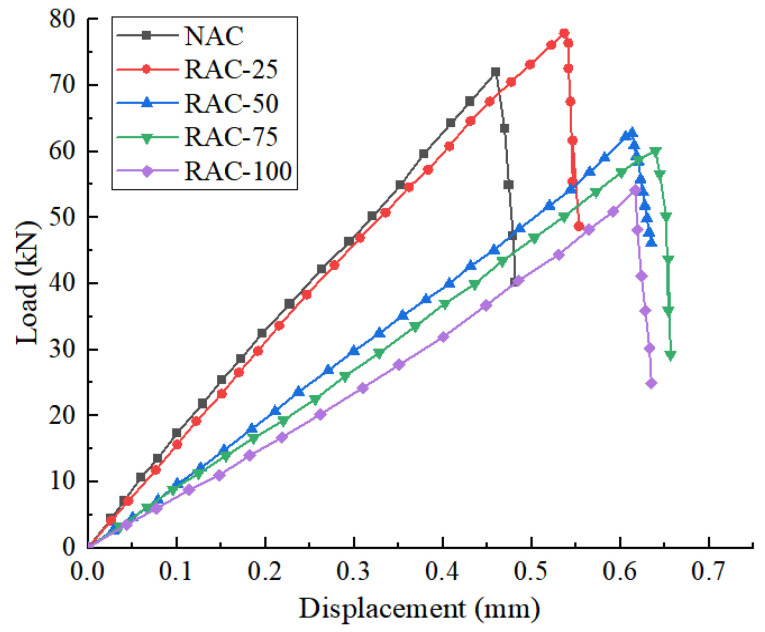
The load–displacement curves of concrete specimens with different RA substitution rates.

**Figure 11:**
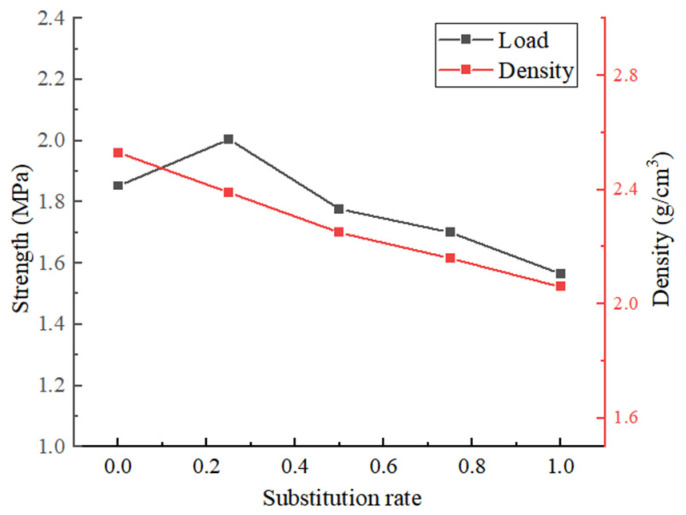
The splitting tensile strength of concrete specimens with different RA substitution rates.

Figure 10 shows the load–displacement curves of the splitting of the specimens. Figure 11 presents the splitting tensile strength of concrete specimens at various rates of recycled aggregate substitution.

It can be seen in the figure that when the replacement rate of recycled coarse aggregate is greater than 25%, the tensile strength of the recycled concrete decreases with the increase in the replacement rate of recycled coarse aggregate. The substitution rate ranges from 25% to 50%, and the tensile strength decreases by 11.5%. When the replacement rate is greater than 50%, the tensile strength of the specimen decreases slowly, and the maximum reduction is only 8.1%. When the replacement rate of recycled coarse aggregate is 25%, the splitting tensile strength of the recycled concrete samples is increased by 8.2% compared with that of the original concrete samples. This is because recycled aggregate has larger porosity and water absorption. Under the premise of full hydration of cement in concrete, a small amount of recycled aggregate will absorb part of the water. The cohesive force between mortar and aggregate increases, which makes the tensile strength of concrete increase. When the replacement rate of recycled aggregate increases gradually, the water absorption of old mortar increases, which hinders the hydration reaction of the cement base, thus reducing the tensile strength of recycled concrete [23].

**Figure 12:**
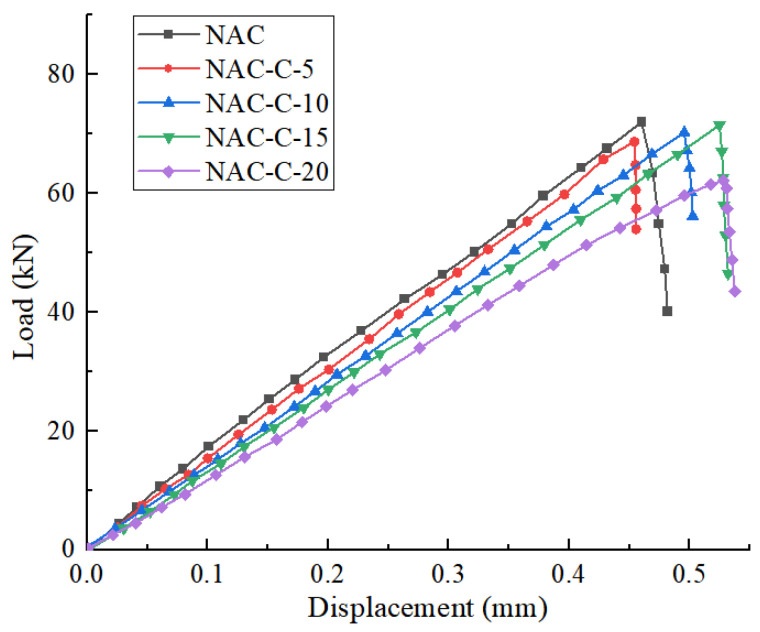
The load–displacement curves of NAC in the splitting tensile test.

**Figure 13:**
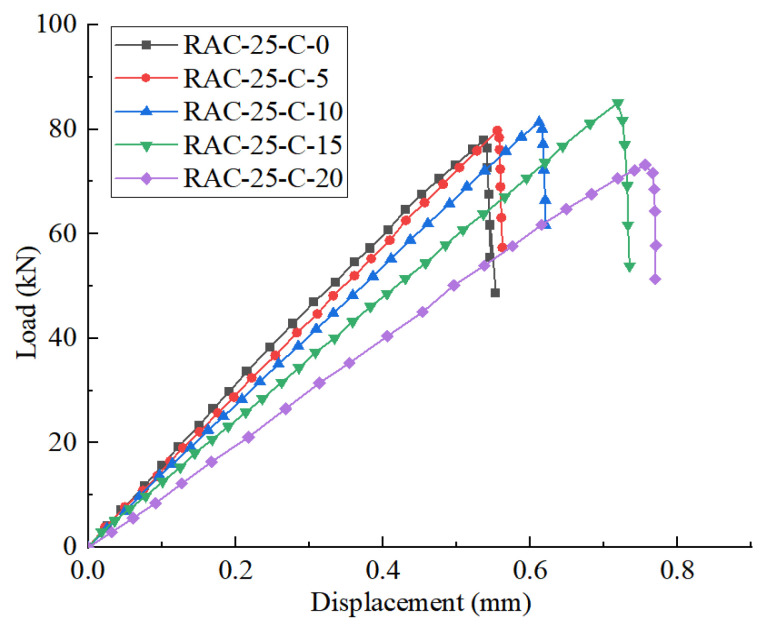
The load–displacement curves of RAC-25 in the splitting tensile test.

Figure 12 and Figure 13 show the load–displacement curves for the split tensile tests of specimens NAC and RAC-25 with varying CGP contents.

It can be observed that when the CGP content is less than 15%, both the NAC and RAC-25 specimens exhibit only two stages in the split tensile failure: elastic deformation and fracture. When the CGP content reaches 20%, both the recycled concrete specimens (RAC-25-C-20) and the native concrete specimens (NAC-C-20) exhibit a yield stage. Prior to the yield point, the load–displacement curve shows a linear increase in load, and after the yield point, the curve displays a brief period of plastic deformation. However, even at this stage, the specimens still undergo brittle failure when the load reaches the ultimate load. This indicates that with a CGP content of 20%, it is possible to improve the ductility of the concrete to some extent.

**Figure 14:**
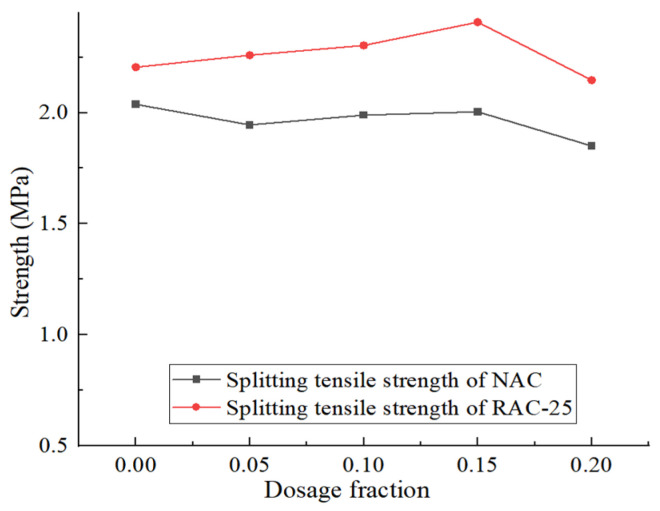
The splitting tensile strength of RAC-25 and NAC.

The splitting tensile strength of RAC-25 and NAC is shown in Figure 14. For concrete specimen RAC-25, when the CGP content increases from 0 to 15%, the splitting tensile strength of the specimen increases slightly with the increase in the CGP content. When the content reaches 15%, it reaches its peak value, at which point the splitting tensile strength of the specimen is 7.9% higher than that of non-coal-gangue-powder-added specimen RAC-25. However, after this point, the splitting tensile strength of the specimen sharply decreases with the increase in the CGP content. When the content reaches 20%, the splitting tensile strength of the specimen is 2.9% lower than that of the RAC-25 specimen.

This indicates that the appropriate addition of CGP can enhance the splitting tensile strength of recycled concrete. A small amount of CGP can fill the micro-pores in concrete, which helps increase its density and reduce porosity. Furthermore, the silicate and aluminate substances in CGP can react with the calcium hydro-silicates in water and cement, producing cementitious materials. This contributes to the improvement in the concrete’s tensile strength. However, when the content is excessive, CGP can increase the water demand of concrete, leading to a higher water–cement ratio, which may result in expansion or delamination in the concrete, negatively affecting its strength.

**Figure 15:**
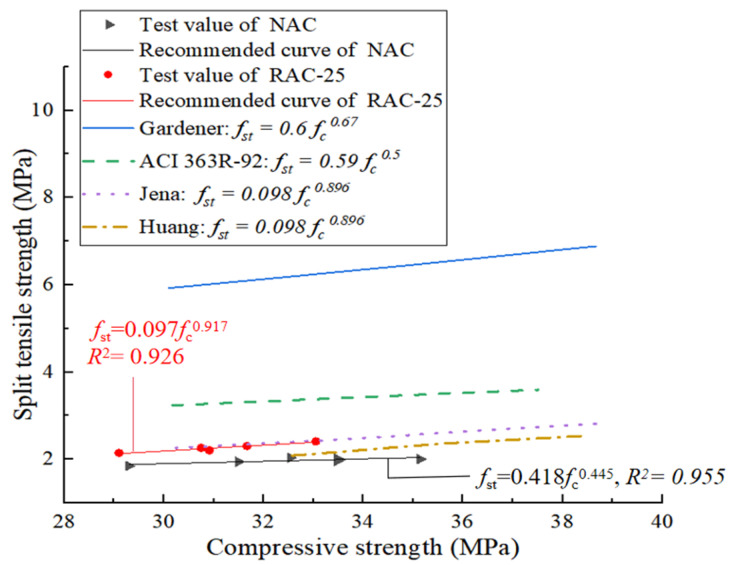
Prediction model of strength and compressive strength.

The tension–compression ratio, which is the ratio of splitting tensile strength to compressive strength of concrete, is the main indicator to measure the brittleness of concrete. Many scholars have performed research on the relationship between the tension and compression ratio of concrete. Based on a large number of experimental data, a variety of analytical models have been proposed, as shown in Figure 15. Based on the experimental data of CGP recycled concrete, the relationship between splitting tensile strength and compressive strength is given by the least squares method. Formula 1 and Formula 2 are the prediction models of the original concrete NAC and the recycled concrete RAC-25, respectively. The formula proposed in this study shows the same trend as the existing prediction model. The models proposed by Gardener [24] and ACI 363R-92 [25] are higher than the test values, while the prediction equations provided by Jena [26] are closer to the test values in this study.

*f*_st_ = 0.097 · *f*_c_^0.917^ (1)

*f*_st_ = 0.418 · *f*_c_^0.445^ (2)

where *f*_st_ is the splitting tensile strength of the specimen and *f*_c_ is the compressive strength of the specimen.

### 3.3. Modulus Analysis

**Figure 16:**
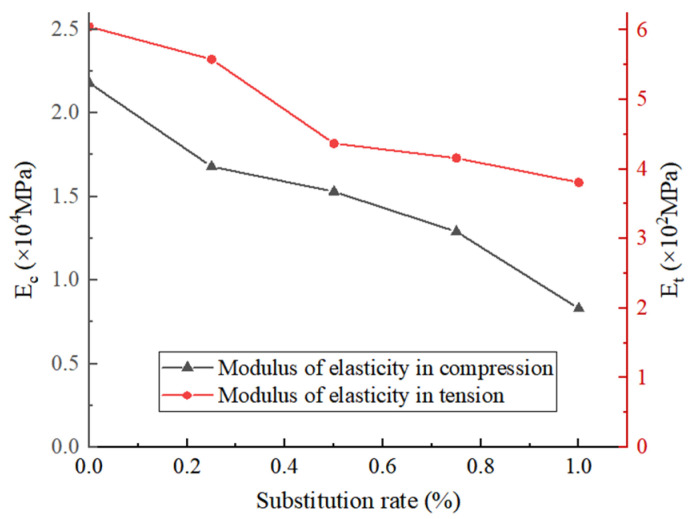
The modulus of the specimens.

The elastic modulus E is the ratio of the stress and the corresponding strain of the material in the elastic deformation stage. According to different stress conditions, there are corresponding values of the tensile elastic modulus (E_t_), the compressive elastic modulus (E_c_), the shear elastic modulus, and the bulk elastic modulus. The curve in Figure 16 reflects the variation in the elastic modulus of the recycled concrete under different coarse aggregate replacement rates.

It can be seen that Et and Ec show a gradual downward trend with the increase in the recycled coarse aggregate content. When the replacement rate of recycled coarse aggregate increases from 0 to 50%, Et decreases by 37.2%, and when the replacement rate is greater than 50%, Et changes tend to be gentle. It can be seen that when the replacement rate of recycled coarse aggregate increases from 0 to 50%, the ability of the concrete to resist tensile deformation decreases sharply because when the content of recycled coarse aggregate is less than 50%, there are fewer internal cracks and pores in the recycled concrete. Under the action of tensile stress, cracks are generated inside the specimen, and the cracks continue to develop and penetrate, and, finally, a fracture occurs. When the content of recycled aggregate is more than 50%, there are many micro-cracks between the old mortar and the gravel in the concrete. Under the action of tensile stress, the micro-cracks deteriorate rapidly, and the old mortar and gravel are peeled off, which greatly reduces the strain of recycled concrete.

**Figure 17:**
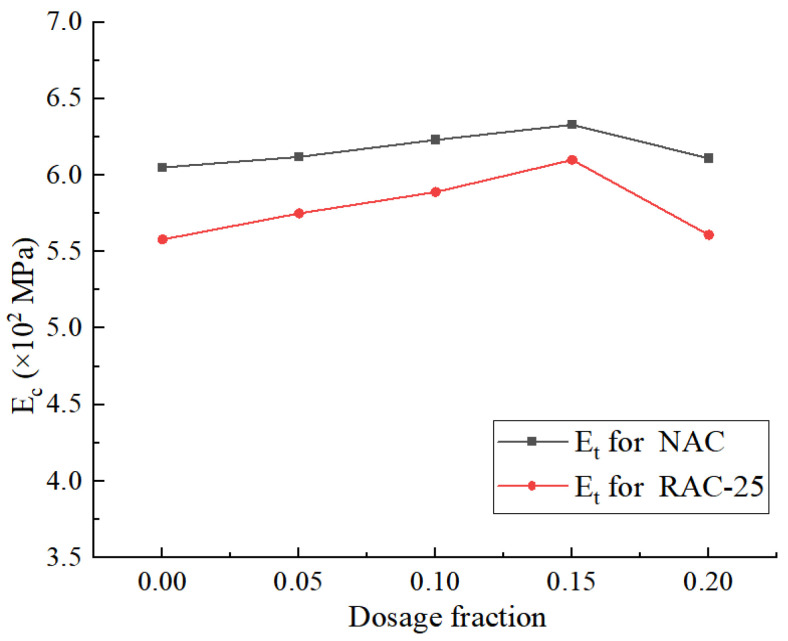
The tensile modulus of the NAC and RAC-25 specimens with different CGP contents.

As shown in Figure 17, the variation trend in the tensile modulus (E_t_) of both the native concrete specimens (NAC) and the recycled concrete specimens (RAC-25) concerning the CGP content is essentially the same.

When the content is less than 15%, the tensile modulus of the NAC specimens increased by only 3.3%. This suggests that the improvement effect of CGP on the tensile modulus of native concrete (NAC) is not very significant. In contrast, the RAC-25 specimens exhibited a more notable increase of 8.5% in the tensile modulus at higher content levels. This is because, in recycled concrete, there are more micro-cracks in the interface transition zone and more voids in old mortar blocks. The silicate and aluminate substances in CGP react with the calcium hydroxide in water and cement, forming cementitious materials. These cementitious materials can effectively fill the gaps and voids, which helps improve the tensile modulus of concrete. Coal gangue powder consists of fine particles, and when the content is too high, the gel-like material formed in the early stages can encase the particles, hindering their reaction with calcium hydroxide. This, in turn, reduces the content of cementitious materials, leading to a sharp decrease in the tensile modulus of concrete.

**Figure 18:**
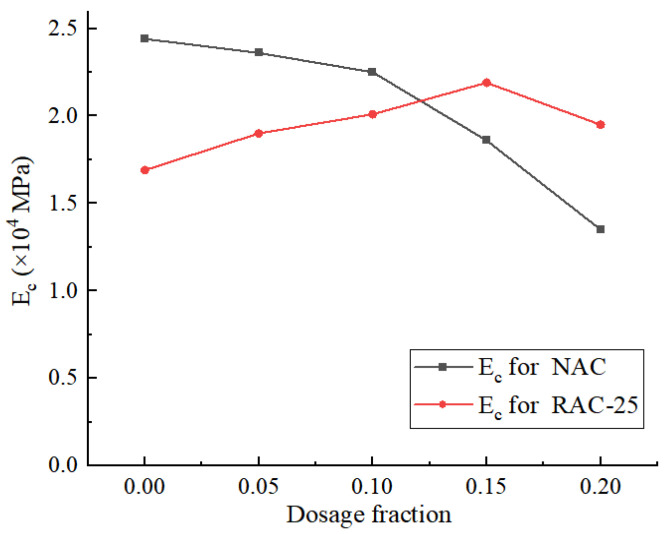
The compression modulus of the NAC and RAC-25 specimens with different CGP contents.

Figure 18 illustrates the variation trend in the compressive modulus (E_c_) for the NAC and RAC-25 specimens with respect to the CGP content.

In the case of the native concrete specimens, the compressive modulus decreases as the CGP content increases. When the content is less than 10%, the decrease in the compressive modulus is relatively gradual. However, when the content exceeds 10%, the rate of decrease becomes more pronounced. When the content reaches 20%, the compressive modulus decreases by 45% compared to the specimens without CGP.

In contrast, the variation trend for the RAC-25 specimens is significantly different from the NAC specimens. When the CGP content is less than 15%, the compressive modulus of the RAC-25 specimens notably increases with the increase in the content. At a content of 15%, the compressive modulus increases by 29.8%. However, when the content exceeds 15%, a decreasing trend becomes apparent.

It is evident that the influence of CGP on the tensile modulus of the NAC and RAC-25 specimens is limited, while it has a significant impact on the compressive modulus.

## 4. Macroscopic Destructive Characteristics

**Figure 19:**
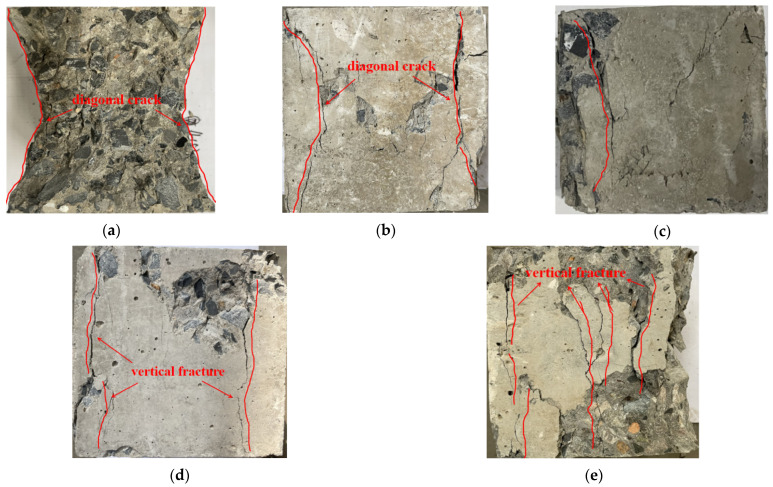
Failure characteristics of concrete specimens at peak strength under different regenerated aggregate replacement rates: (**a**) specimen NAC, (**b**) specimen RAC-25, (**c**) specimen RAC-50, (**d**) specimen RAC-75, and (**e**) specimen RAC-100.

Under different replacement rates of coarse aggregate, the macroscopic failure characteristics of the specimen at the peak strength in the uniaxial compression test are different (Figure 19).

The test results show that the crack development law and failure form of the recycled concrete test block are different from those of the ordinary concrete. When the RCA content is 0 and 25%, the failure characteristics of the specimens are basically the same. At the initial stage of loading the specimens, with the increase in the load, the stress in the recycled concrete specimens gradually increases because the constraint effect of the loaded pad produces a boundary effect, resulting in small transverse deformation and the largest transverse expansion deformation in the middle of the specimens. As the load continues to increase, cracks begin to appear in the specimens. The first vertical crack is close to the side surface of the specimens, extends upward and downward along the oblique direction, and gradually turns to the corner of the specimens, forming an “eight” shape crack connected upside down and, finally, forming two quadrigonal cones failure surfaces with upper and lower symmetry.

When the RCA content gradually increases, the failure mode of the specimens changes obviously. At the initial stage of the cracking of the specimens, a vertical main crack appears in the middle part near the edge of the free surface on both sides, and with the increase in the load, the crack extends along the vertical direction to the upper and lower sides. Meanwhile, several irregular short cracks appear on the free surface of the specimens. When the load increases to the peak strength of the specimens, the short surface cracks are connected, the middle concrete bulges outward, and the surface concrete begins to bulge and spall outward. At this time, the concrete around the main crack is severely broken, and the integrity of the specimens after failure is significantly reduced.

**Figure 20:**
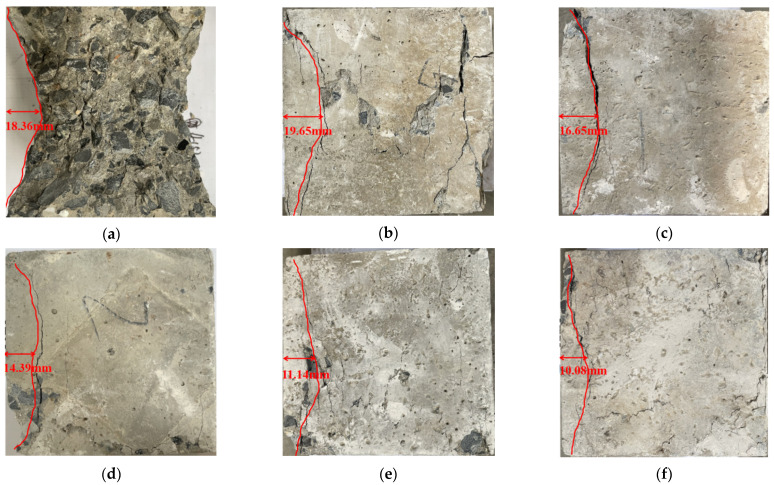
The failure characteristics of RAC-25 with different contents of CGP at peak strength: (**a**) specimen NAC, (**b**) specimen RAC-25, (**c**) specimen RAC-25-C-5, (**d**) specimen RAC-25-C-10, (**e**) specimen RAC-25-C-15, and (**f**) specimen RAC-25-C-20.

Figure 20 reflects the failure characteristics of the recycled concrete specimens at peak strength under different CGP contents.

It can be seen that the failure modes of NAC, RAC-25, and RAC-25-C-5 are basically the same, and the main crack form has not changed significantly. When the content of CGP is more than 5%, the failure mode of the specimens begins to change. As the content of CGP increases gradually, the inclination angle of the crack in the specimens gradually increases. When the content reaches 20%, it is approximately a vertical crack. In addition, with the increase in the CGP content, the position of the main crack is closer to the edge of the free surface, which is different from the failure characteristics of NAC and RAC-25. The integrity of the specimens mixed with CGP is relatively good after reaching the peak strength. Except for the main crack penetration, the development of other small cracks is not obvious. It can be seen from this that when CGP is added, the composition and structure of the recycled concrete change, the failure mechanism changes, and the failure mode changes.

## 5. Mesoscopic Cracks

**Figure 21:**
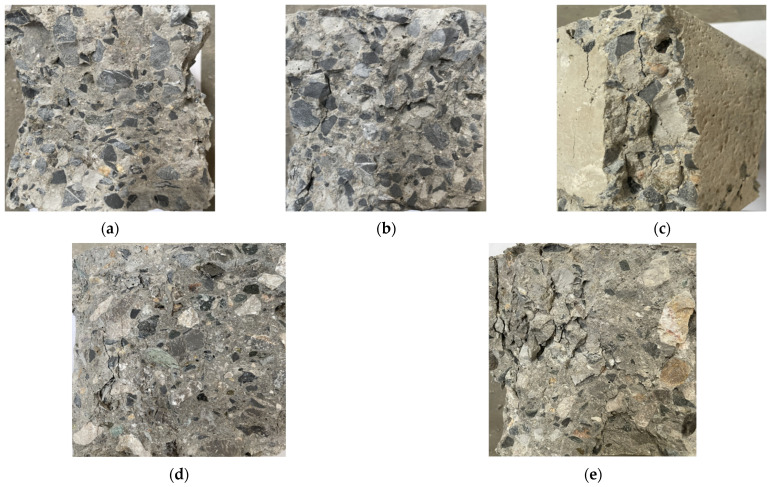
The failure characteristics of concrete specimens after failure under different recycled aggregate replacement rates: (**a**) specimen NAC, (**b**) specimen RAC-25, (**c**) specimen RAC-50, (**d**) specimen RAC-75, and (**e**) specimen RAC-100.

The analysis of the micro-cracks after the failure of the specimens is shown in Figure 21.

The gravel is surrounded by old mortar to form recycled concrete aggregate, and three interfaces are formed at the same time, which are the gravel–old mortar interface (GOI), the gravel–new mortar interface (GNI), and the new–old mortar interface (ONI). After the failure of the natural aggregate concrete cube specimens, the failure parts are mainly concentrated in the interface transition zone (ITZ) between the natural coarse aggregate and the cement mortar. However, for the recycled concrete, the development of cracks after damage is different. The damage is mainly manifested in two parts: one is the interfacial transition zone between recycled coarse aggregate and cement mortar, and the other is the recycled coarse aggregate itself (damaged from the middle of the recycled aggregate). In addition, after the compressive test of the recycled aggregate concrete, the mortar of the recycled coarse aggregate is easier to separate, and the interfacial transition zone between the recycled coarse aggregate and the mortar is more obvious. In terms of crack width, the cracks between the recycled aggregate and the natural mortar after the destruction of recycled concrete are wider than those after the destruction of the ordinary concrete.

**Figure 22:**
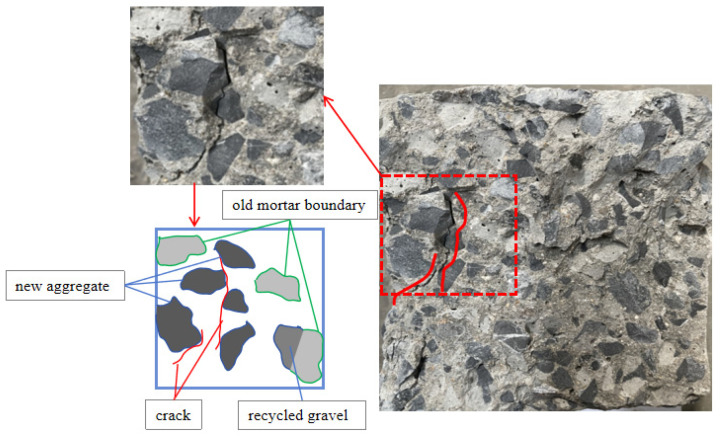
Microscopic cracks inside specimen RAC-25.

Figure 22 shows the internal crack distribution of specimen RAC-25 after failure. It can be seen that the crack appears at the interface between the new gravel aggregate and the new mortar, and the trend of the crack extends along the surface of the new gravel to the new mortar and finally forms a through crack. However, there are no obvious cracks on the surface of both the old mortar block and the recycled aggregate; in addition, there are no cracks on the old mortar block and GOI.

**Figure 23:**
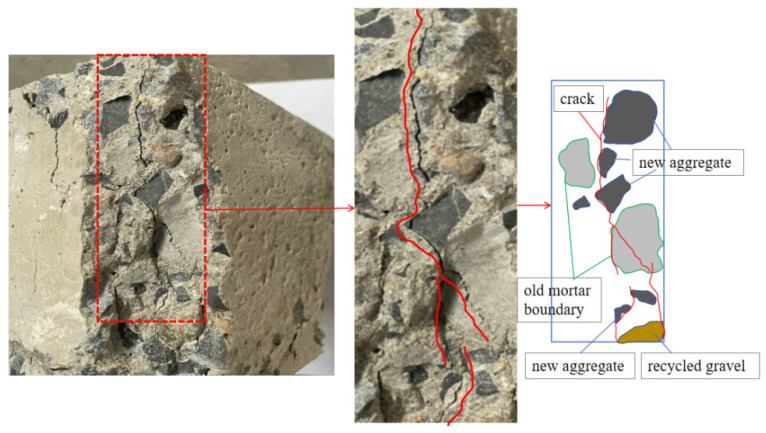
Microscopic cracks inside specimen RAC-50.

Compared with the RAC-25 specimen, the crack distribution of the RAC-50 specimen after failure has changed significantly, as shown in Figure 23.

Firstly, the location of the cracks changes greatly. It can be found that the internal cracks of RAC-50 are distributed in the gravel–new mortar interface (GNI), the new–old mortar interface (NOI), the recycled gravel–new mortar interface (RGNI), and the old mortar block. Secondly, the morphology of the cracks is also significantly different. In specimen RAC-50, the cracks are mainly long-through. Some of the cracks extend from the GNI interface to the ONI interface, forming long-through cracks. The other part of the cracks starts from the crack initiation position of the ONI interface and runs through the entire old mortar block, causing the old mortar block to break and extend to the crack initiation position of the GNI interface, forming a through crack. In addition, the density and width of the cracks in RAC-50 are significantly higher than those in RAC-25.

**Figure 24:**
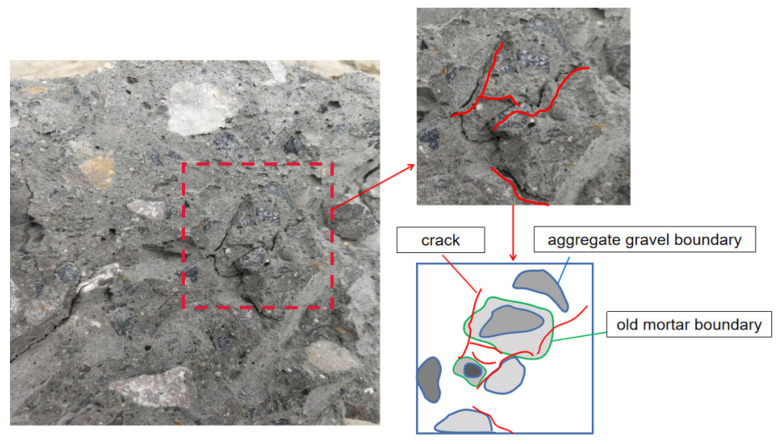
Microscopic cracks inside specimen RAC-75.

Figure 24 shows the internal crack distribution of RAC-75 when it is damaged. The recycled concrete is composed of aggregate gravel, old mortar, and new mortar.

The specimen is damaged internally under the action of load, and cracks begin to occur at the weak mechanical properties. By analyzing the cracking law of recycled concrete, it can be found that the cracks are mainly concentrated at GOI and ONI, and the cracking degree at the interface between new and old mortar (ONI) is the most obvious. The distribution characteristics of cracks are mainly in two forms. First, the crack width is evenly distributed along the contact surface of the new mortar and the old mortar. The other is that the crack is perpendicular to the contact surface through the new mortar and the old mortar, and the crack width becomes narrower from the old mortar to the new mortar. It can be judged that when the recycled concrete is damaged, the old mortar first cracks, and as the load increases, the crack extends to the new mortar.

From the failure mode of RAC-100 in Figure 21e, it can be seen that when the replacement rate of coarse aggregate with a particle size of 10–20 mm reaches 100%, the degree of fragmentation after the failure of the specimen is larger, the degree of peeling of the mortar attached to the aggregate surface is more obvious, and the mortar near each crack is mostly in a peeling state. By comparing the RAC-75 test piece, it can be seen that when the replacement rate of coarse aggregate with a particle size of 10–20 mm is 75% and 100%, the cracks formed after the failure of the test piece are mainly short cracks, and the cracks are mainly distributed in the new–old mortar interface (ONJ), the recycled gravel–new mortar interface (RGNJ), and the old mortar block. Among them, the new–old mortar interface (ONJ) and the internal cracks of the old mortar block account for a large proportion.

## 6. Crack Analysis

**Figure 25:**
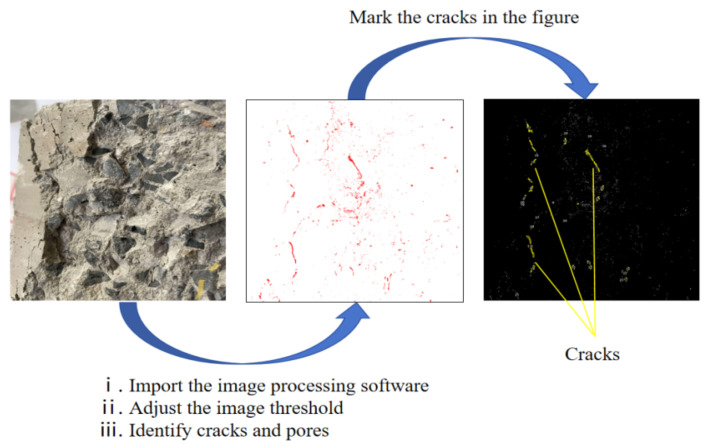
Gray image of damaged concrete.

The distribution law of internal cracks after concrete failure can reflect the failure mechanism and mechanical characteristics of concrete to a certain extent. Digital image processing software was used to identify the pores and cracks in the image, and the cracks in the image were marked so as to obtain the distribution characteristics of cracks and the proportion of cracks. Figure 25 shows the process of calculating the crack ratio.

Firstly, the image was converted into a grayscale image, and the appropriate grayscale value was selected to mark the cracks in the image. Then, the grayscale ratio corresponding to the cracks was measured. The number of pixels in the image was divided by the total number of pixels in the image. The ratio obtained is the ratio of the crack area.

**Figure 26:**
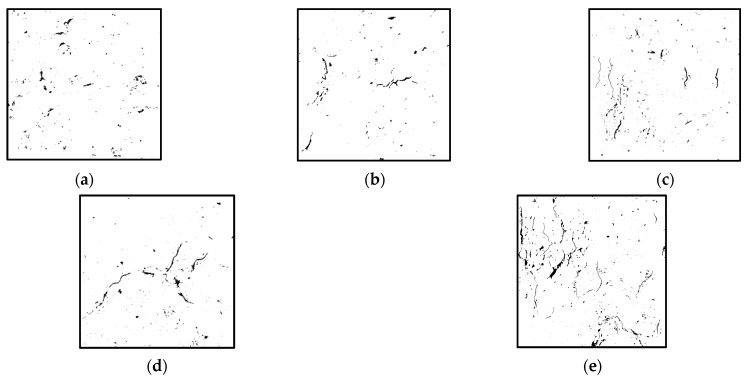
Gray maps of pores and cracks of recycled concrete under different replacement rates: (**a**) specimen NAC, (**b**) specimen RAC-25, (**c**) specimen RAC-50, (**d**) specimen RAC-75, and (**e**) specimen RAC-100.

Figure 26 shows the gray images of the cracks and pores in the recycled concrete under different replacement rates of 10–20 mm aggregates.

It can be seen that with the increase in the aggregate replacement rate, the proportion of pore density and crack area of the specimens increased significantly. For the original NAC concrete, the number of internal cracks is small after the failure of the specimen, and most of them are short cracks. The distribution of pores in the specimen is relatively uniform, and the stress distribution in the concrete is also relatively uniform. The failure mode is a pressure-type failure, which is consistent with the failure mode of the specimen analyzed above. When the recycled aggregate is added, the internal cracks of the specimen increase obviously under the action of the load. When the internal stress of the specimen reaches the peak strength, the cracks inside the specimen continue to expand under the action of tensile stress and, finally, damage occurs.

**Figure 27:**
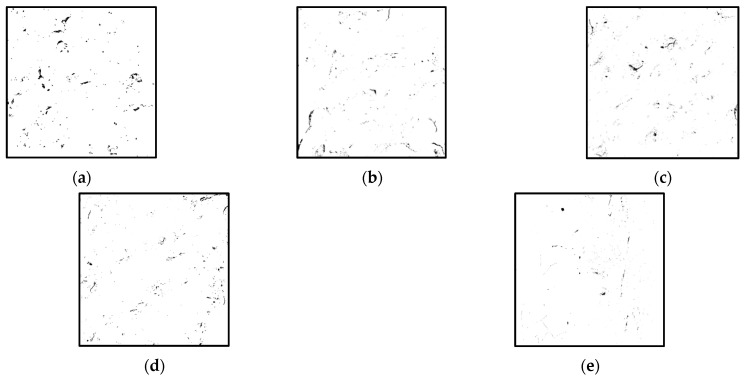
Gray images of pores and cracks of primary concrete with different CGP contents: (**a**) specimen NAC-C-0, (**b**) specimen NAC-C-5, (**c**) specimen NAC-C-10, (**d**) specimen NAC-C-15, and (**e**) specimen NAC-C-20.

Figure 27 shows the grayscale images of the cracks and pores of the NAC original concrete under different CGP contents.

It can be seen that under different CGP contents, the distribution density of the pores and cracks in the original concrete specimen changes little, and its distribution is relatively uniform. For the original NAC concrete, the crack density after failure is significantly smaller than that of the recycled concrete specimen. With the increase in the content of CGP, the gray value of the internal pores and cracks after the failure of the specimen gradually decreases, and the particle size of the pores and the width of the cracks of the specimen obviously decrease.

**Figure 28:**
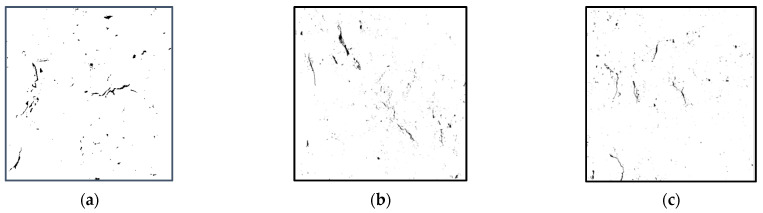

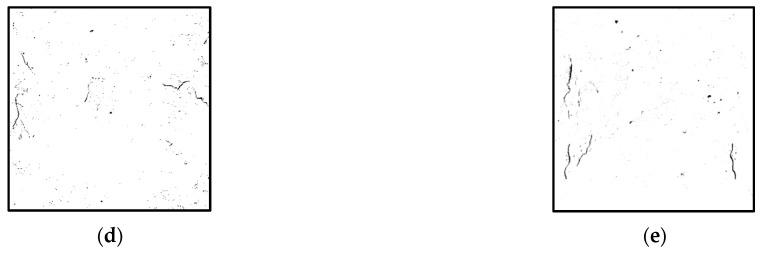
Gray images of pores and cracks of recycled concrete with different CGP contents: (**a**) specimen RAC-25-C-0, (**b**) specimen RAC-25-C-5, (**c**) specimen RAC-25-C-10, (**d**) specimen RAC-25-C-15, and (**e**) specimen RAC-25-C-20.

Figure 28 shows the gray images of the cracks and pores of the RAC-25 recycled concrete specimen.

It can be seen that when the content of CGP increases from 0 to 15%, the area ratio of cracks after specimen failure decreases slightly, and the width of cracks decreases obviously. When the content reaches 20%, the width of the crack becomes obviously larger, and the position of the crack gradually approaches the edge. In addition, the distribution of pores near the fracture is relatively dense, which suggests that the density of pores directly affects the generation of fractures. This is because when the specimen is subjected to load, stress concentration occurs at the pores, resulting in tiny cracks. As the load increases, the cracks at the pores continue to expand and form a penetrating main crack. When the stress at the crack reaches the peak strength, the specimen is destroyed.

**Figure 29:**
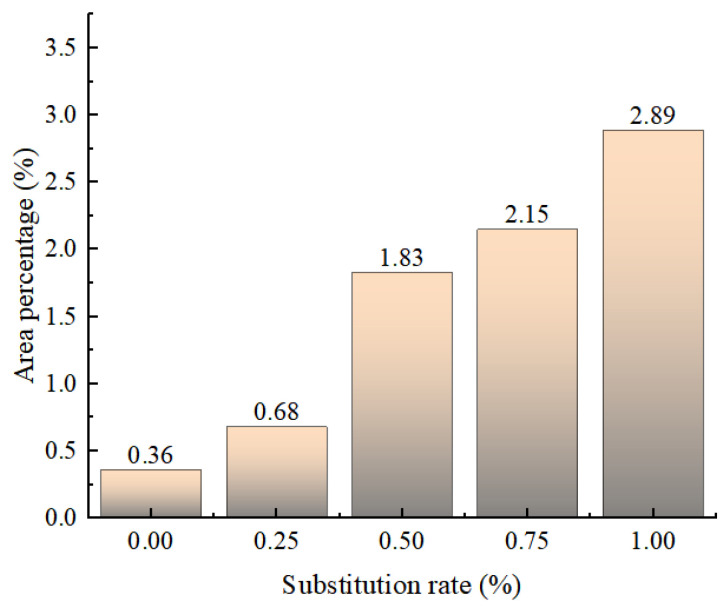
The proportion of cracks in recycled concrete under different aggregate replacement rates.

Through comprehensive analysis, it can be seen that the density of internal pores and cracks after concrete failure will increase with the increase in the replacement rate of 10–20 mm recycled coarse aggregate. At the same time, the proportion of cracks also gradually increases. As shown in Figure 29, when the replacement rate of recycled aggregate exceeds 25%, the proportion of cracks increases sharply, increasing by nearly 1.7 times.

**Figure 30:**
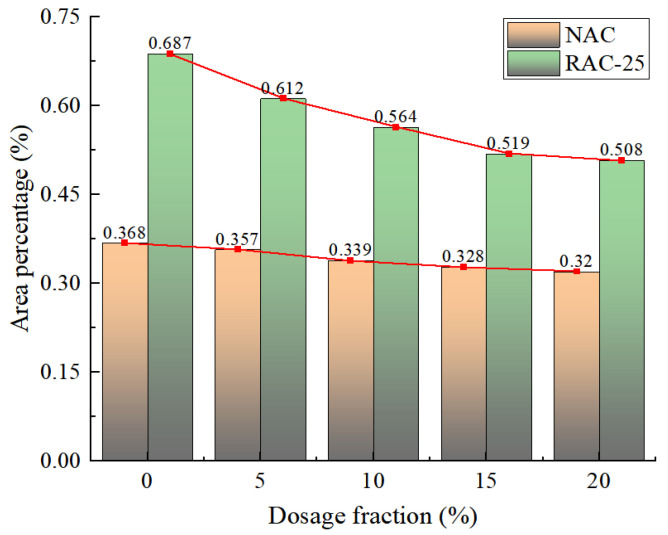
The proportion of concrete cracks under different CGP contents.

When the concrete is not mixed with CGP, the density of internal pores and cracks after the failure of the specimen is significantly greater than that of the specimen mixed with CGP, and the crack width is larger. With the increase in the CGP content, the proportion of cracks in the original concrete NAC specimens gradually decreases, but the change range is relatively gentle. When the content exceeds 15%, it reaches stability. At this time, the proportion of cracks is 0.38%, which is 9% lower than that of the NAC specimen. For recycled concrete specimen RAC-25, after adding CGP, the proportion of cracks obviously decreases with the increase in the CGP content (Figure 30).

When the content with CGP is 15%, the proportion of cracks is 0.519%, which is 23.5% lower than that of the RAC-25 specimen. Similar to the NAC specimens, when the content exceeds 15%, the proportion of cracks tends to be stable.

## 7. Discussion

The shape, particle size, porosity, surface structure, and apparent density of concrete aggregate have a great influence on the mechanical properties of recycled aggregate concrete. The strength of recycled concrete is reduced, which is mainly caused by the porosity of the concrete because of the porous structure of recycled coarse aggregate. Because the recycled coarse aggregate has porosity, the water transport process in the interfacial transition zone will be relatively smooth, allowing it to reach the concrete to produce a more hydration process [27]. Therefore, the water absorption capacity of recycled aggregate is very strong, which will lead to uneven water absorption of the natural aggregate around it, resulting in a decrease in the splitting strength and compressive strength of recycled concrete.

There are different bonding surfaces in all kinds of concrete. There are two kinds of bonding surfaces in ordinary concrete. One is the direct bonding surface between natural aggregate and mortar, and the other is the bonding surface composed of natural aggregate and natural aggregate [28]. However, the difference is that in the interior of recycled concrete, there are many corners, and the surface of the recycled coarse aggregate is adhered to cement stones and mortar. Therefore, the internal bonding surface of recycled concrete will be more complex, and various bonding surfaces will appear. Recycled concrete has cement, sand, natural aggregate, and recycled aggregate as the main components of the polymer. Therefore, there are three different bonding surfaces in recycled concrete, which are the bonding surface between the natural aggregate and the bonding mortar on the recycled aggregate, the direct bonding surface between the old mortar and the new mortar on the recycled aggregate, and the direct bonding surface between the new mortar and the natural aggregate. Previous studies have shown that for the interface between natural aggregate and bonding mortar, the direct interface between the new mortar and the natural aggregate has little effect on the compressive strength of recycled concrete [29]. However, the direct bonding between the old mortar and the new mortar bonded on the recycled aggregate is faced with re-bonding. The compressive strength of raw concrete has a great influence.

According to the uniaxial compression test results of the specimens in this paper, when the replacement rate of recycled coarse aggregate increases gradually, the compressive strength and compressive elastic modulus of the specimens decrease, and the decrease in E_s_ is greater than that of compressive strength. The phenomenon that the compressive strength of recycled concrete decreases with the increase in the aggregate replacement rate can be attributed to the weakening of the interface between the new and old mortar and the evolution of the pore structure. The porosity of the old mortar attached to the surface of the recycled aggregate is high, and the secondary bonding with the fresh mortar forms a loose old and new mortar interface. As the substitution rate increases, the proportion of new and old mortar interfaces increases, resulting in a decrease in stress transfer efficiency. This is because the hydration products (such as C-S-H gel) at the interface between the new and old mortars are sparsely distributed, and micro-cracks are preferentially initiated here, eventually leading to penetrating damage [30].

The water absorption characteristics of recycled aggregate lead to a local increase in the effective water–binder ratio in fresh concrete, forming a non-uniform hydration structure. When the substitution rate is greater than 25%, the elastic modulus decreases significantly (the elastic modulus of RAC-50 is 37.2% lower than that of NAC), indicating that the stiffness of the material deteriorates because of the increase in porosity. In addition, the plastic strain decreases with the increase in the replacement rate (the plastic strain of RAC-100 is only 63% of that of NAC, indicating that the brittle characteristics of old mortar limit the ductile deformation ability of concrete).

When the content of CGP is 15%, the micro-powder filling effect can reduce the porosity of the interface between the new and old mortars, and the active SiO_2_ and Ca(OH)_2_ react to generate additional C-S-H gel (Formula (3)) to strengthen the interface bonding [31].

SiO_2_ + Ca(OH)_2_ + H_2_O→C-S-H (3)

This mechanism makes the peak strength of RAC-25-C-15 increase by 6.9%, and the strain capacity (ε_p = 0.41%) is better than that of the sample without CGP (plastic strain = 0.35%).

## 8. Conclusions

The purpose of this study is to explore the comprehensive utilization of recycled concrete aggregate and coal gangue powder in solid waste materials. This study is conducted from two aspects. On the one hand, it studies the change in the basic mechanical properties of recycled concrete and the failure characteristics of samples under different aggregate replacement rates. On the other hand, the influence of CGP on the mechanical properties of recycled concrete is studied. Through a single-factor test and theoretical analysis, the macroscopic failure characteristics and mesoscopic failure mechanism of recycled concrete under different CGP contents are obtained, and the strength change characteristics and failure characteristics of concrete under different recycled aggregate replacement rates are obtained. The main conclusions are as follows:

1. The peak strength of recycled concrete decreases with the increase in the replacement rate of 10–20 mm recycled coarse aggregate. When the replacement rate is 25%, the decrease in strength is the smallest. When the content of CGP increases from 0 to 20%, the peak strength of recycled concrete increases first and then decreases. When the content is 15%, the peak strength reaches its maximum.
2. The density of internal pores and cracks after concrete failure will increase with the increase in the replacement rate of 10–20 mm recycled coarse aggregate. At the same time, the proportion of cracks also increases gradually. When the replacement rate of recycled aggregate reaches 50%, the proportion of cracks increases sharply, increasing by nearly 1.7 times.
3. When CGP is added to concrete, the density of the internal pores and cracks after the failure of the specimen is obviously reduced. With the increase in the CGP content, the proportion of cracks in the original concrete specimens does not decrease significantly. When the content exceeds 15%, it decreases by 9% compared with specimen NAC, while the proportion of cracks in recycled concrete specimen RAC-25 decreased significantly with the increase in the CGP content. When the content of CGP was 15%, the proportion of cracks decreased by 23.5%.
4. CGP has a significant effect on the failure mode of recycled concrete. As the content of CGP gradually increases, the position of the main crack is closer to the edge of the free surface, which is significantly different from the specimen mixed with CGP. The integrity of the specimens mixed with CGP is relatively good after reaching the peak strength. Except for the main crack penetration, the development of other small cracks is not obvious.

## Figures and Tables

**Table 1:** Composition of materials.

Type	CaO	SiO_2_	Al_2_O_3_	Fe_2_O_3_	MgO
Cement	57.64	25.34	5.67	3.42	3.12
CGP	0.62	47.44	25.5	5.10	1.55

**Table 2:** Basic properties of coarse aggregates.

Type	Size/mm	Water Content/%	Water Absorption/%	Crush Value/%	Bulk Density/(kg/cm^3^)	Apparent Density/(kg/cm^3^)
RA	5–20	2.3	5.5	16.14	1250.7	2433.2
NA	5–20	0.34	0.53	12.10	1545.6	2796.4

**Table 3:** Mix proportion.

Specimen Name	W/C	Cement (kg)	Fine Aggregate (kg)	CA			CGP (kg)
				NA (5–10 mm) (kg)	NA (10–20 mm) (kg)	RA (10–20 mm) (kg)	
NAC	0.426	357	633	384.3	896.7	-	-
RAC-25	0.426	357	633	384.3	672.5	224.2	-
RAC-50	0.426	357	633	384.3	448.4	448.4	-
RAC-75	0.426	357	633	384.3	224.2	672.5	-
RAC-100	0.426	357	633	384.3	-	896.7	-
NAC-C-5	0.426	339.15	633	384.3	896.7	-	17.85
NAC-C-10	0.426	321.3	633	384.3	896.7	-	35.7
NAC-C-15	0.426	303.45	633	384.3	896.7	-	53.55
NAC-C-20	0.426	285.6	633	384.3	896.7	-	71.4
RAC-25-C-5	0.426	339.15	633	384.3	672.5	224.2	17.85
RAC-25-C-10	0.426	321.3	633	384.3	672.5	224.2	35.7
RAC-25-C-15	0.426	303.45	633	384.3	672.5	224.2	53.655
RAC-25-C-20	0.426	285.6	633	384.3	672.5	224.2	71.4

## Data Availability

The data used to support the findings of this study are available from the corresponding author upon request.
